# Intranasal inoculation of an MVA-based vaccine induces IgA and protects the respiratory tract of hACE2 mice from SARS-CoV-2 infection

**DOI:** 10.1073/pnas.2202069119

**Published:** 2022-06-09

**Authors:** Jeffrey L. Americo, Catherine A. Cotter, Patricia L. Earl, Ruikang Liu, Bernard Moss

**Affiliations:** ^a^Laboratory of Viral Diseases, National Institute of Allergy and Infectious Diseases, NIH, Bethesda, MD 20892

**Keywords:** COVID-19, coronavirus vaccine, vaccinia virus, mucosal immunity, respiratory immunity

## Abstract

Current vaccines have greatly diminished the severity of the COVID-19 pandemic, even though they do not entirely prevent infection and transmission, likely due to insufficient immunity in the upper respiratory tract. Here, we compare intramuscular and intranasal administration of a live, replication-deficient modified vaccinia virus Ankara (MVA)–based Severe Acute Respiratory Syndrome Coronavirus 2 (SARS-CoV-2) spike (S) vaccine to raise protective immune responses in the K18-hACE2 mouse model. Using a recombinant MVA expressing firefly luciferase for tracking, live imaging revealed luminescence of the respiratory tract of mice within 6 h and persisting for 3 d following intranasal inoculation, whereas luminescence remained at the site of intramuscular vaccination. Intramuscular vaccination induced S-binding–Immunoglobulin G (IgG) and neutralizing antibodies in the lungs, whereas intranasal vaccination also induced Immunoglobulin A (IgA) and higher levels of antigen-specific CD3^+^CD8^+^IFN-γ^+^ T cells. Similarly, IgG and neutralizing antibodies were present in the blood of mice immunized intranasally and intramuscularly, but IgA was detected only after intranasal inoculation. Intranasal boosting increased IgA after intranasal or intramuscular priming. While intramuscular vaccination prevented morbidity and cleared SARS-CoV-2 from the respiratory tract within several days after challenge, intranasal vaccination was more effective as neither infectious virus nor viral messenger (m)RNAs were detected in the nasal turbinates or lungs as early as 2 d after challenge, indicating prevention or rapid elimination of SARS-CoV-2 infection. Additionally, we determined that neutralizing antibody persisted for more than 6 mo and that serum induced to the Wuhan S protein neutralized pseudoviruses expressing the S proteins of variants, although with less potency, particularly for Beta and Omicron.

## Significance

Despite the ability of current vaccines to significantly prevent severe disease due to Severe Acute Respiratory Syndrome Coronavirus 2 (SARS-CoV-2), vaccinated individuals are still susceptible to infection and contribute to the spread of the virus. The present study demonstrates that a live, replication-deficient recombinant virus vaccine induces greater immunity and a greater level of protection in the respiratory tract of susceptible transgenic mice when inoculated intranasally compared with intramuscularly. Second-generation vaccines administered via the upper respiratory tract have the potential to limit the spread of SARS-CoV-2 more effectively than current vaccines.

The rapid development of SARS-CoV-2 vaccines was a stunning achievement that is contributing to the control of the COVID-19 pandemic. Several types of vaccines—including mRNA, adenovirus-vectors, recombinant spike (S) protein, and inactivated Severe Acute Respiratory Syndrome Coronavirus 2 (SARS-CoV-2)—have demonstrated the ability to protect against severe disease. Nevertheless, these vaccines, which are administered systemically, reduce but do not prevent virus infection and transmission, and therefore approaches that provide further immunity are desirable (1). SARS-CoV-2 spreads by droplet and aerosol so that the nasal and oral mucosa are the first barriers to infection. In general, the intranasal (IN) route of vaccination induces greater mucosal immunity compared with the intramuscular (IM) route. An example is the live, attenuated influenza virus vaccine, called LAIV or FluMist, which is approved as a nasal spray in some countries. Unlike inactivated influenza vaccine, LAIV induces nasal Immunoglobulin A (IgA) and CD8^+^ T cells (2). Similarly, IN administration of adenovirus-vectored SARS-CoV-2 vaccines reduce viral loads in upper and lower respiratory tracts following challenge in several animal models (3–6) and an aerosolized vaccine appeared safe and immunogenic in a phase I trial (7), although a trial of another adenovirus-based nasal spray vaccine was discontinued because of low immunogenicity (https://ir.altimmune.com/news-releases/news-release-details/altimmune-announces-update-adcovidtm-phase-1-clinical-trial). Studies of IN vaccination with additional vectors are needed.

Modified vaccinia virus Ankara (MVA) is a highly attenuated, replication-defective, immunogenic smallpox vaccine strain that is undergoing clinical testing as a vector for multiple pathogens (8) as well as SARS-CoV-2 (www.clinical trials.gov). Although usually administered IM or subcutaneously, several reports have shown that MVA-based vectors induce protective mucosal and systemic immune responses when administered IN to animals (9–13). In addition, combined IM and IN vaccination of camels with an MVA-based vaccine reduced excretion of Middle East respiratory syndrome (MERS)-CoV, although the efficacy of IN alone was not reported (14).

The present study was initiated to extend previous demonstrations of the ability of IM administered MVA-vectored vaccines to protect against SARS-CoV-2 challenge in animal models (15–18). We previously reported (15) that IM injection of MVA expressing a modified S protein with mutations that stabilized the prefusion form, inactivated the furin cleavage site, and deleted the endoplasmic retention signal induced a type 1 immune response with neutralizing antibody and CD8^+^IFN-γ^+^ T cells, and protected K18-hACE2 transgenic mice from respiratory infection with SARS-CoV-2. In addition, passive transfer of serum from vaccinated mice to unvaccinated mice protected them from lethal SARS-CoV-2 infection. Here, we show persistence of neutralizing antibody and protection of transgenic hACE2 mice for more than 6 mo after one or two IM inoculations with an MVA-based modified S protein vaccine. However, whereas IM vaccination induced Immunoglobulin G (IgG) neutralizing antibodies and cleared infection of the respiratory tract, IN inoculations also induced IgA antibodies in the lungs and blood, and after two IN vaccinations neither SARS-CoV-2 nor subgenomic (sg) mRNAs were detected in the nasal turbinates or lungs at 2 or 5 d after challenge. IN delivery of a live recombinant vaccine has the potential to reduce infection and transmission of SARS-CoV-2.

## Results

### Relationship of IM Vaccination Dose to Immune Response.

**Fig. 1.**
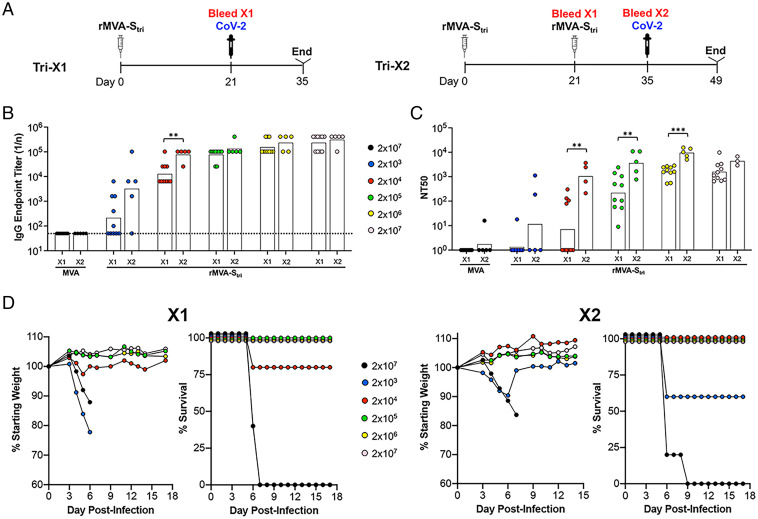
Effects of rMVA-S_tri_ dose on immune responses and protection. (*A*) K18-hACE2 mice were vaccinated IM once (Tri-X1, *n* = 10) or twice (Tri-X2, *n* = 5) with 2 × 10^3^ to 2 × 10^7^ PFU of rMVA-S_tri_ or 2 × 10^7^ PFU of MVA as a control and bled 21 d after the first vaccination and 14 d after the second. The mice were challenged IN with 10^5^ TCID_50_ of SARS-CoV-2 following the first or second collection of blood. (*B*) SARS-CoV-2 S-binding IgG antibodies in sera following first and second vaccinations. Endpoint titers in sera of 10 mice vaccinated once and 5 mice vaccinated twice were determined by ELISA with immobilized SARS-CoV-2 S. Titers of sera from individual mice and geometric means are shown. The dotted line represents the limit of detection. (*C*) Pseudovirus neutralizing titers of the same serum samples in *B* were determined and plotted as NT_50_ of individual mice and geometric means. (*D*) Weights and survival of mice vaccinated 1× and 2× with indicated doses of rMVA-S_tri_ or MVA are shown. Significance was determined by nonparametric Mann–Whitney test. **P < 0.01, ***P = 0.0007.

In our previous study (15), we determined that one or two IM injections of 2 × 10^7^ PFU of a recombinant MVA (rMVA-S_tri_) expressing a SARS-CoV-2 S protein that had been triply modified by stabilization of the prefusion structure, inactivation of the furin cleavage site, and deletion of the endoplasmic retention signal, induced S-binding and neutralizing antibodies and protected transgenic K18-hACE2 mice from lethal respiratory infection with the Wuhan strain of SARS-CoV-2 (15). To correlate vaccine dose with antibody level and protection against a lethal SARS-CoV-2 challenge, we varied the dose of rMVA-S_tri_ from 2 × 10^3^ to 2 × 10^7^ PFU administered once (1×) or again (2×) at the same dose 3 wk later (Fig. 1*A*). In this experiment and subsequent ones, mice immunized with the parental MVA served as controls. Serum antibodies were analyzed 3 wk after the prime and 2 wk after the boost. At the lowest dose of 2 × 10^3^ PFU of rMVA-S_tri_, half of the mice had higher ELISA S-binding titers than the control mice after the prime and nearly all had elevated titers after the boost (Fig. 1*B* and Dataset S1). With higher doses of rMVA-S_tri_, binding antibodies were detected in all mice after the prime and, except for the dose of 2 × 10^4^ PFU, the endpoint titers were similar after the boost compared with the prime. In addition, the boosted values following inoculation of 2 × 10^4^ PFU or higher were similar to each other.

A G protein-deficient vesicular stomatitis (rVSVΔG) vector bearing the S protein of the Wuhan strain of SARS-CoV-2 was used for pseudovirus assays to detect neutralizing antibody (19) unless mentioned otherwise in the figure legends. For mice receiving one or two injections of 2 × 10^3^ PFU of rMVA-S_tri_, none of 10 and 2 of 5, respectively, had 50% neutralizing antibody titers (NT_50_s) above 25, the limit of detection (Fig. 1*C* and Dataset S1). For mice that received 2 × 10^4^ PFU of rMVA-S_tri_, approximately half had positive neutralizing titers after one vaccination and all had positive titers after the second. Boosting significantly increased the NT_50_s except at the highest dose of 2 × 10^7^. However, the boosted values for all doses were similar to each other.

To correlate antibody titers with protection, the mice were challenged IN with a lethal dose of 10^5^ TCID_50_ of SARS-CoV-2 (Wuhan strain) at 3 wk after the first vaccination or 2 wk after the second. Mice that received a single inoculation with 2 × 10^3^ PFU succumbed by day 6 or 7, whereas a majority that were boosted with the same dose survived (Fig. 1*D* and Dataset S1). With one exception, all mice that received one or two inoculations of 2 × 10^4^ PFU or higher survived with no weight loss. Taking all the data together, survival correlated with an NT_50_ of ∼100 and single doses of 2 × 10^6^ PFU and boosts of 2 × 10^5^ PFU or higher exceeded this by 10- to 100-fold (Dataset S1). The relatively low level of neutralizing antibody correlating with protection is consistent with passive antibody transfer experiments carried out in our previous study in which the NT_50_ in recipient mice of 160 was fully protective (15).

### Duration of Protective Immunity after IM Vaccination.

**Fig. 2.**
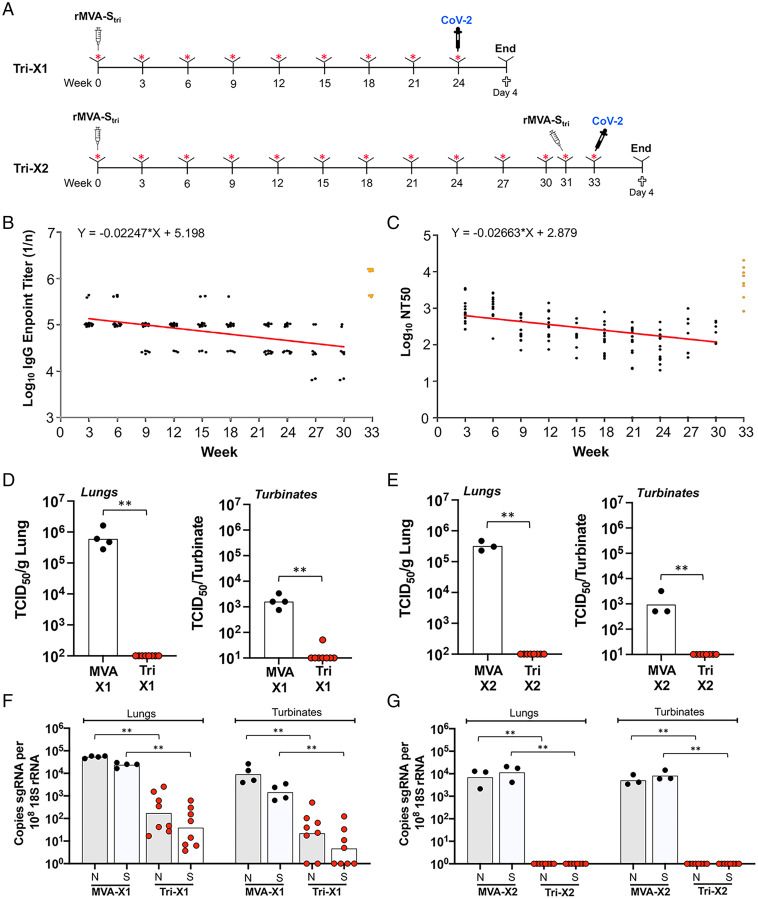
Duration of immune response and protection following IM vaccination. (*A*) K18-hACE2 mice were vaccinated once (Tri-X1, *n* = 8) or twice (Tri-X2, *n* = 8) with 2 × 10^7^ PFU of rMVA-S_tri_ or MVA as a control once (*n* = 4) or twice (*n* = 3) and bled at 3-wk intervals. The mice were challenged IN with 10^5^ TCID_50_ SARS-CoV-2 at week 24 after the first vaccination or at week 2 after the second vaccination, which was administered 31 wk after the first, and killed 4 d later. (*B*) SARS-CoV-2 S-binding IgG antibodies were determined by ELISA and endpoint titers of sera from individual mice from 3 to 30 wk following the single vaccination (black dots) and the samples obtained 2 wk after the second vaccination (orange dots) were plotted. The trend line (red) was determined by linear regression of log transformed geometric mean values for the 30 wk following the first vaccination. (*C*) Lentivirus pseudoviral neutralizing titers of sera from individual mice were determined and plotted as NT_50_ and color-coded as in *B*. The trend line was determined as in *B*, excluding values below the limit of detection. (*D*) Infectious SARS-CoV-2 in lungs and nasal turbinates was analyzed at 4 d after challenge of mice that had been vaccinated once (1×). Individual and geometric mean TCID_50_ are shown. (*E*) Same as *D*, except mice were vaccinated twice (2×). (*F*) The sgN (shaded) and sgS (unshaded) RNAs in the lungs and nasal turbinates of mice vaccinated 1× were determined by ddPCR and normalized to 18S rRNA. Values for individual mice and the geometric means are shown. (*G*) Same as *F*, except that mice were vaccinated 2×. Significance: **P ≤ 0.006.

To determine the duration of protective immunity, the SARS-CoV-2 S-binding and pseudoviral neutralizing titers were monitored over a 6- to 8-mo period following IM injection of 2 × 10^7^ PFU of rMVA-S_tri_ (Fig. 2*A*). The decline in binding antibody titer was 19% at 4 wk and 75% over 6 mo (Fig. 2*B*). Similarly, the neutralizing antibody titer declined 22% at 4 wk and 81% over 6 mo (Fig. 2*C*) but remained above the level that correlated with protection. At 31 wk after the initial vaccination, some mice received a second dose of rMVA-S_tri_, resulting in over a log increase in binding and neutralizing titers (Fig. 2 *B* and *C*). The NT_50_ was significantly higher (*P* = 0.0013; two-tailed *t* test, nonparametric, Mann–Whitney) than obtained with the second vaccination 3 wk after the first, as determined in the previous section, supporting the advantage of delaying a booster dose when appropriate.

Mice that received only a single vaccination with rMVA-S_tri_ or control MVA were challenged 24 wk later with SARS-CoV-2. High virus titers were detected in lungs and turbinates on day 4 of mice immunized with the control MVA, whereas little to no infectious virus was detected in the lungs or nasal turbinates of mice that received a single immunization with rMVA-S_tri_ (Fig. 2*D*). It is possible, however, that TCID_50_ assays overestimate the reduction in virus replication because of neutralization of virus by antibodies during tissue homogenization. Determination of sgRNAs avoids this potential problem, and distinguishes mRNAs synthesized during replication from input viral genomic RNA. The sgN and sgS RNAs on day 4 were quantified by digital droplet (dd) PCR. To compensate for variations in the amounts of tissue recovered, the sgRNAs were normalized to 18S ribosomal RNA in the same sample. High levels of sgRNAs were detected in the lungs and nasal turbinates of mice that received the parental MVA but were significantly lower by 2 to 3 logs in mice that had received a single vaccination with the rMVA-S_tri_ 6 mo prior to challenge (Fig. 2*F*).

Mice that were boosted at week 31 and challenged at week 33 also had no detectable virus in the lungs or turbinates (Fig. 2*E*). In addition, sgRNAs were not detected 4 d after challenge in the mice that received a second vaccination (Fig. 2*G*). We concluded that neutralizing antibodies persisted for more than 6 mo after a single immunization and that mice exhibited strong immunity to challenge. Furthermore, a second vaccination after 31 wk boosted neutralizing antibody and provided a higher level of protection.

### Infection of the Respiratory Tract by rMVAs Administered IN.

**Fig. 3.**
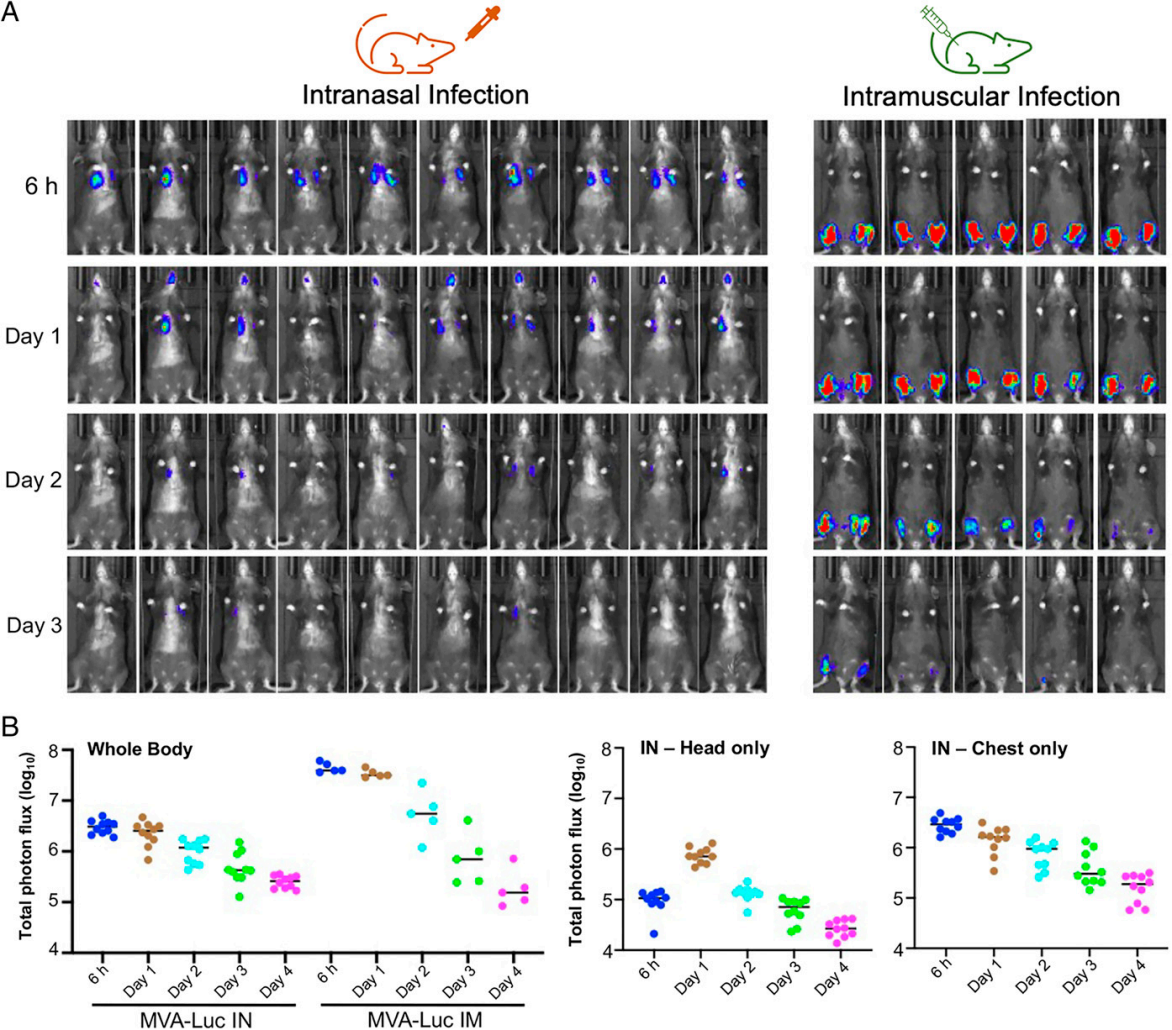
Live animal imaging of Luc following IN or IM inoculation of rMVA. (*A*) Mice (C57BL/6) were inoculated with 2 × 10^7^ PFU of MVA/Luc IN (*n* = 10) or IM (*n* = 5), the latter divided into each thigh. At the indicated hours and days, luciferin was injected intraperitoneally, and Luc was detected with a live animal imager. The exposure time and binning factor were kept constant. Ventral views of mice are shown. Intensity increases from blue to red. (*B*) Total photon flux (photons per cm^2^/sr) was determined for the whole body or head and chest separately. Data are representative of two independent experiments.

Although rMVA-based vaccines are usually administered systemically, there have been a few studies in which an rMVA vector was given IN to mice, but the extent and duration of expression had not been determined. An rMVA that expresses firefly luciferase (MVA/Luc) was previously used to monitor IM vaccination of BALB/c mice (20). In that study, live imaging showed dose-dependent luminescence at the site of inoculation on day 1 that gradually declined over the next few days. Here, we compared IN and IM inoculation with MVA/Luc of C57BL/6 mice, which have the genetic background of K18-hACE2 mice used for protection studies. At 6 h after inoculation, the detection of luminescence in the chest indicated infection of the lower respiratory tract (Fig. 3*A*). At 24 h, luminescence increased in the head but began to diminish in the chest (Fig. 3). Luminescence in both head and neck continued to decrease over the next few days. The 50-µL inoculum administered to lightly anesthetized mice likely led to direct infection of the lungs as well as the nares. Following IM inoculation, high luminescence at the site of injection was detected at 6 and 24 h and then gradually diminished (Fig. 3). Because of quenching, the intensity of luminescence detected is inversely affected by tissue depth, and therefore the values obtained after IN and IM are not directly comparable. The decline in luminescence over a few days and the absence of detectable spread from the inoculation sites are consistent with the inability of MVA to replicate in mammals and is an important safety feature.

### Immune Responses in the Lungs of IN- and IM-Vaccinated Mice.

**Fig. 4.**
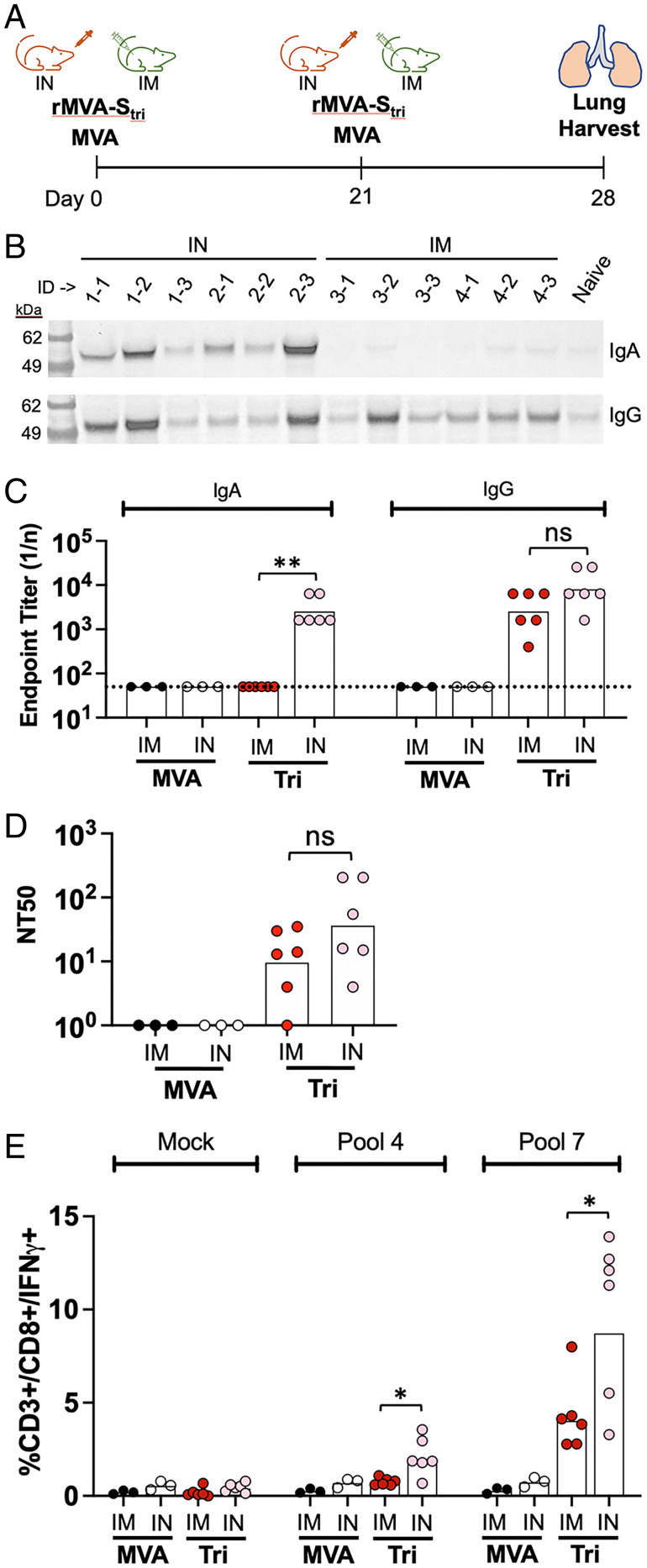
Immune responses in the lungs of vaccinated mice. (*A*) K18-hACE mice were vaccinated with 2 × 10^7^ PFU of rMVA-S_tri_ IN (*n* = 6) or IM (*n* = 6) and then boosted 2 wk later via the same route. The mice were killed 1 wk after the boost and perfused lungs were excised, dissociated, and centrifuged to collect supernatant and cells. (*B*) Lung tissue supernatants of vaccinated mice and a naïve mouse were analyzed by SDS/PAGE and blots were probed with anti-IgA and anti-IgG antibodies. Positions of marker proteins in kilodaltons shown on left. (*C*) IgA and IgG SARS-CoV-2 S binding titers of the lung tissue supernatants were determined by ELISA. Values for individual mice and geometric mean are shown. The dotted line represents the limit of detection. (*D*) Pseudovirus neutralizing titers were determined on lung tissue supernatants. Values for individual mice and geometric mean titers are shown. (*E*) Lung cells were mock stimulated or stimulated with peptide pool 4 or 7 and the percent CD3^+^CD8^+^IFN-γ^+^ were determined by flow cytometry; 10,000 to 70,000 events were collected for each sample. Significance: *P ≤ 0.04, **P = 0.002; ns, not significant.

The ability of MVA to infect the upper and lower respiratory tracts following IN inoculation suggested that the vaccine could induce a local immune response. To evaluate this prediction, K18-hACE2 mice were vaccinated with rMVA-S_tri_ by the IN or IM route (Fig. 4*A*). The mice received two vaccinations by the same route 3 wk apart, were killed 1 wk after the second, and antibodies present in supernatants of lung tissue after dissociation and removal of cells determined (21). Both IM and IN vaccinations induced IgG antibodies compared with an unvaccinated control, but only IN induced IgA as shown by Western blotting (Fig. 4*B*). Specific binding of IgG and IgA to SARS-CoV-2 S protein was determined by ELISA (Fig. 4*C*). Although similar levels of S-specific IgG were induced by IN and IM vaccinations, only the IN induced S-specific IgA. Like the IgG, the neutralizing antibody levels in the lung homogenates were also not significantly different for the IN and IM inoculations (Fig. 4*D*). Thus, the presence of IgA was a distinctive feature of IN vaccination.

By scanning a SARS-CoV-2 S peptide pool library for the ability to stimulate CD8^+^ T cells from the blood of vaccinated BALB/c and C57BL/6 mice, we previously documented strong stimulation by pool 7, which contains peptides of the receptor binding domain (RBD), and weak stimulation by pool 4, which contains peptides of the N-terminal domain. These two pools were used to stimulate lung cells from K18-hACE2 mice in the present study and the percent CD3^+^CD8^+^IFN-γ^+^ cells were enumerated by flow cytometry. Both IN and IM vaccinations with MVA-S_tri_ increased the CD3^+^CD8^+^IFN-γ^+^ cells stimulated by peptide pool 7 compared with the parental MVA (Fig. 4*E*). The CD3^+^CD8^+^IFN-γ^+^ cells comprised ∼10% of the CD3^+^ cells from the lungs of mice vaccinated IN compared with ∼4% of cells from mice vaccinated IM. Fewer CD8^+^IFN-γ^+^ cells were stimulated with peptide pool 4 compared with pool 7 from mice vaccinated IN and few or none were detected from lungs of mice vaccinated IM. Taken together, these data indicate that IN inoculation of rMVA-S_tri_ leads to induction of more IgA and CD8^+^ T cells than IM inoculation, but to similar levels of IgG and neutralizing antibody.

### Enhanced Protection of the Respiratory Tract following IN Vaccination.

**Fig. 5.**
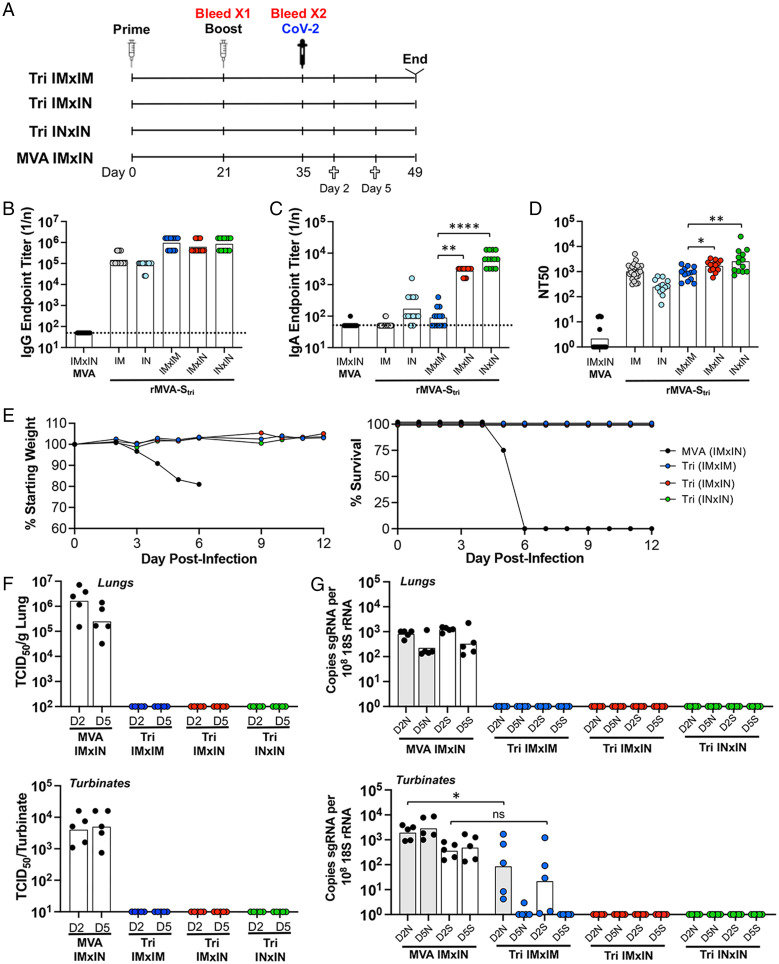
Comparison of immune responses and protection following IN and IM vaccinations. (*A*) K18-hACE2 mice were primed and boosted with rMVA-S_tri_ by IN or IM route to form three groups of 13 mice: IMxIM, IMxIN, and INxIN. In addition, a control group received MVA IM followed by MVA IN. Mice from each group were bled at 3 wk after the prime and 2 wk after the boost. At the latter time, mice were challenged by IN inoculation of 10^5^ TCID_50_ of SARS-CoV-2. On days 2 and 5 after challenge, five mice from each group were killed and three remained to follow weight and morbidity. (*B* and *C*) Serum IgG and IgA endpoint ELISA titers are shown for individual mice and the geometric mean. The dashed line represents the limit of detection. (*D*) Individual and geometric mean pseudovirus neutralizing titers are shown. (*E*) Three mice of each group were monitored for weight loss and survival. (*F*) At days 2 and 5 after challenge with SARS-CoV-2, homogenates were prepared from the lungs and nasal turbinates of five mice from each group and the TCID_50_ of SARS-CoV-2 determined. (*G*) RNA was extracted from the homogenates and copies of sgN (shaded) and sgS (unshaded) RNA were determined by ddPCR and normalized to 18S rRNA in the same sample. Significance: *P = 0.04, **P < 007, ****P < 0.0001; ns, not significant.

To compare the route of vaccination with the extent of protection from a SARS-CoV-2 challenge, K18-hACE2 mice were inoculated IM or IN with rMVA-S_tri_, followed 3 wk later by a second IM (IMxIM) or IN (IMxIN or INxIN) immunization (Fig. 5*A*). As a control, another group of mice was inoculated with the parental MVA given IMxIN. In this experiment, IgG and IgA antibodies were analyzed in serum. The IgG ELISA titers in the three groups were similar after the first injection and were boosted to similar levels by the second (Fig. 5*B*). IgA was detected after the first IN inoculation and boosted by the second (Fig. 5*C*). Although IgA was not detected after one or two IM inoculations, it was induced significantly when the IM inoculation was followed by an IN inoculation. Neutralizing antibodies were induced in each case with the titers only slightly higher after IMxIN and INxIN compared with IMxIM (Fig. 5*D*).

The mice were challenged with SARS-CoV-2 as in the preceding experiments. The mice of each group vaccinated with rMVA-S_tri_ exhibited no signs of weight loss or morbidity, including three held for an extended time, whereas mice immunized with the control MVA lost weight and did not survive (Fig. 5*E*). In the experiment depicted in Fig. 2, we analyzed virus titers in the nasal turbinates and lungs at 4 d after challenge with SARS-CoV-2. Here, we assessed the virus titers at days 2 and 5 after infection; the 2-d date was chosen to determine whether the IN vaccination led to an earlier protective effect compared with IM. Following the SARS-CoV-2 challenge, high virus titers were recovered from the lungs and nasal turbinates of the control MVA-vaccinated mice, whereas none was detected from either the lungs or turbinates of the IMxIM, IMxIN, or INxIN rMVA-S_tri_–vaccinated mice (Fig. 5*F*). Similarly, no sgS or sgN RNAs were detected in the lungs of these mice at 2 or 5 d after challenge (Fig. 5*G*). However, sgN and sgS mRNAs were detected in the nasal turbinates of IMxIM mice on day 2 and in one of five mice on day 5, whereas none was found in mice that received an IMxIN or INxIN immunization on either day (Fig. 5*G*).

The detection of SARS-CoV-2 sgRNAs in the nasal turbinates on day 2 but little or none on days 4 and 5 and none in the lungs on either day following challenge of IM-vaccinated mice indicates that the infection was rapidly suppressed. The absence of detectable sgRNAs in the turbinates or lungs of mice, even at 2 d in mice that were vaccinated IMxIN or INxIN, suggests that infection was even more rapidly eliminated or prevented.

### Two IN Vaccinations Are Better than One.

**Fig. 6.**
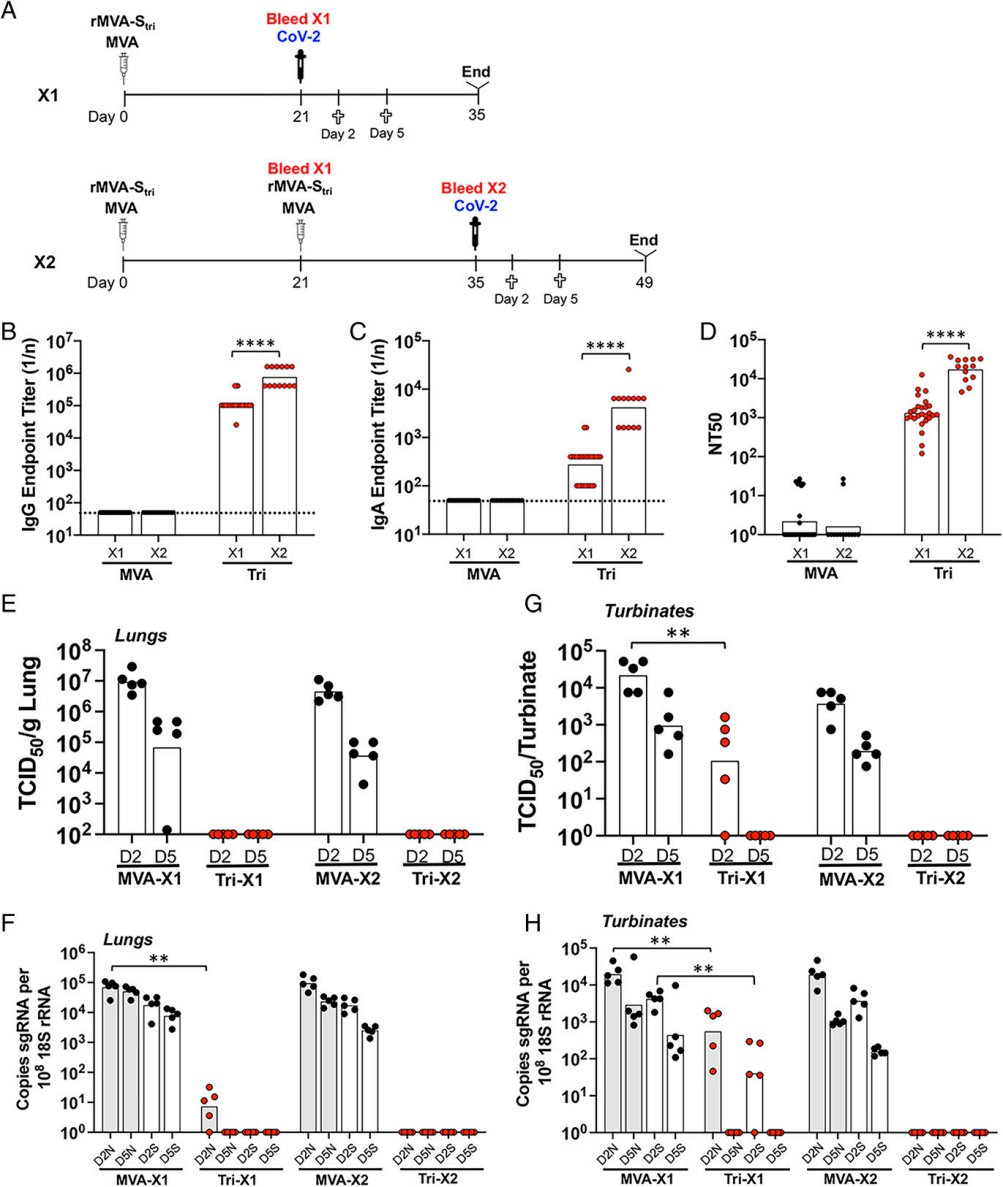
Comparison of immunity to SARS-CoV-2 and protection following one or two IN vaccinations. (*A*) K18-hACE2 mice were inoculated IN with rMVA-S_tri_ once (1×, *n* = 13) or a second time (2×, *n* = 13) after 3 wk. Blood was collected prior to challenge with SARS-CoV-2 3 wk after the first vaccination and 2 wk after the second. At 2 and 5 d after challenge, blood was collected and the animals killed. (*B* and *C*) Serum IgG and IgA endpoint ELISA titers are shown for individual mice and geometric means. The dotted line represents the limit of detection. (*D*) Individual and geometric mean pseudovirus neutralizing titers are shown. (*E*) At days 2 and 5 after challenge with SARS-CoV-2, homogenates were prepared from the lungs of five mice from each group and the TCID_50_ of SARS-CoV-2 determined. (*F*) RNA was extracted from the same lung homogenates as in *E* and the copies of sgN (shaded) and sgS (unshaded) RNA determined by ddPCR and normalized to 18S rRNA in the same sample. (*G*) TCID_50_ determined from nasal turbinates. (*H*) RNA was extracted from same nasal turbinate samples as in *G*. Significance: *P < 0.05, **P, <0.008, ****P < 0.0001.

To confirm and further evaluate the early role of IN vaccination, we compared the relative abilities of one and two IN immunizations with rMVA-S_tri_ to protect K18-hACE2 mice against SARS-CoV-2 challenge (Fig. 6*A*). As in the experiments described above, serum IgG, IgA, and neutralizing antibodies were detected after the first IN immunization and each significantly increased after the second (Fig. 6 *B*–*D*). The mice were challenged with SARS-CoV-2 at 21 d after the prime or 14 d after the boost. Mice that received one or two inoculations with rMVAS_tri_ exhibited little or no weight loss and all survived, whereas all those receiving MVA exhibited severe weight loss and except for one, succumbed to the infection. Considerable infectious SARS-CoV-2 was detected following challenge on days 2 and 5 in the lungs of mice that received the control MVA, whereas none was detected in the mice that received one or two IN immunizations with rMVA-S_tri_ (Fig. 6*E*). The control mice had sgN and sgS RNAs in the lungs on day 2 following challenge, which decreased only slightly on day 5, indicating continued infection (Fig. 6*F*). In mice that received a single vaccination, on day 2 following challenge sgS RNA was undectable and sgN RNA was significantly reduced by about 4 logs in the lungs compared with the control; on day 5 neither sgS nor sgN RNA were detected in the immunized mice (Fig. 6*F*). Neither sgS nor sgN RNA was detected in the lungs on either day following challenge of mice that received two vaccinations, consistent with the higher antibody levels after the second vaccination (Fig. 6*F*).

In the nasal turbinates of mice that received a single vaccination, the virus was about 3 logs lower than in the controls on day 2 after challenge and none was detected on day 5 (Fig. 6*G*). Similarly, there was a significant reduction in sgN and sgS RNAs on day 2 and none was detected on day 5 in mice that received a single vaccination (Fig. 6*H*). However, neither virus nor sgRNAs were detected on either day after challenge in mice that received the second vaccination (Fig. 6 *G* and *H*).

We concluded that a single IN vaccination with rMVA-S_tri_ resulted in significant protection of the upper and lower respiratory tract on day 2 that was complete by day 5, whereas a second IN vaccination entirely prevented the detection of infection even on day 2.

### Neutralization of Pseudoviruses Expressing Variant S Proteins.

**Fig. 7.**
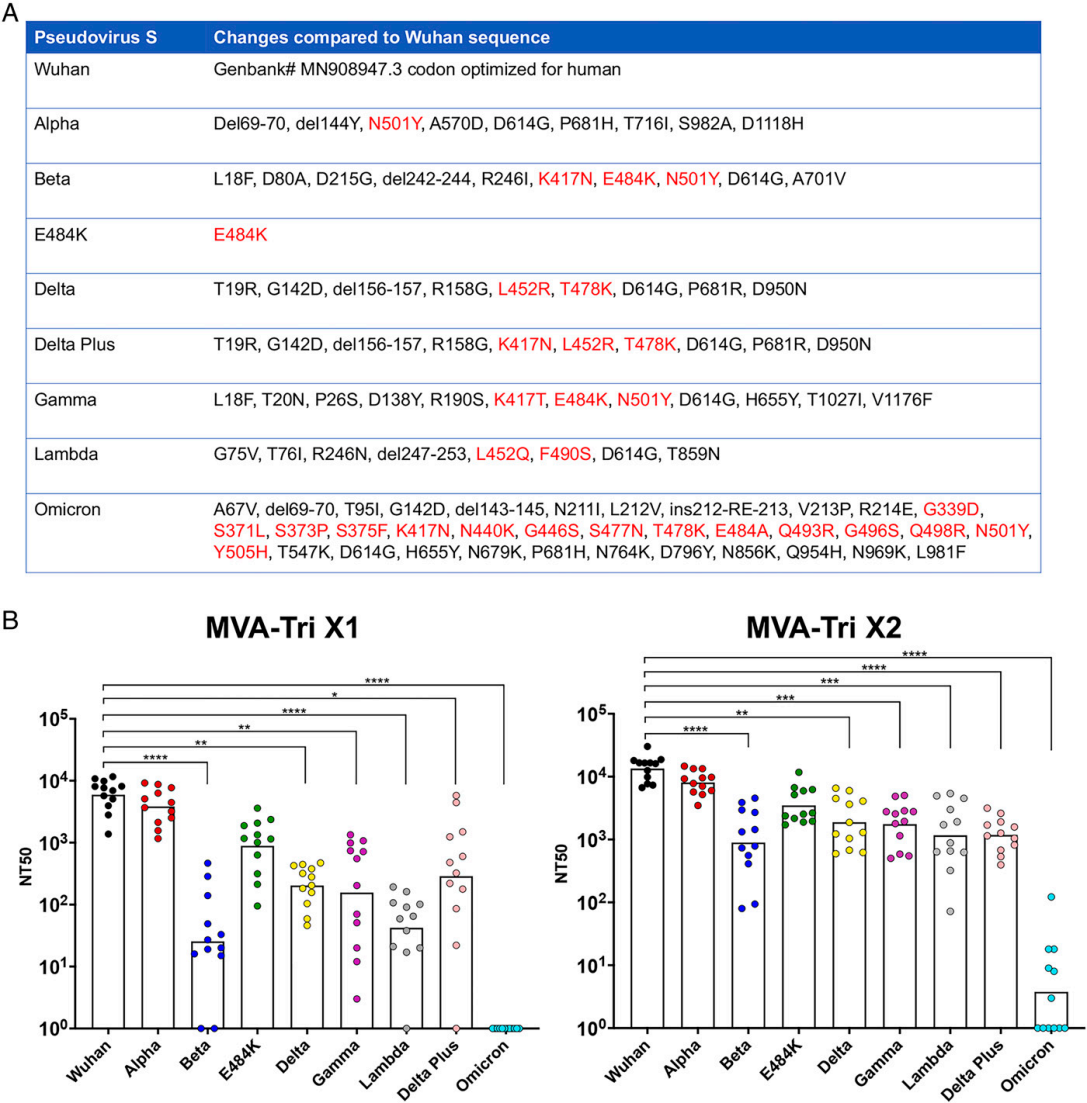
Neutralization of pseudoviruses expressing variant S proteins. (*A*) Amino acid differences in S of SARS-CoV-2 variants relative to that of Wuhan are shown. Amino acids within the RBD are colored red. Abbreviations: del, deleted; ins, inserted. (*B*) C57BL/6 were vaccinated IM with 2 × 10^7^ PFU of rMVA-S_tri_ and bled 3 wk later. At the latter time, the mice were boosted and bled after 2 wk. Serum from individual mice obtained after the first (1×) and second (2×) immunizations were tested for the ability to neutralize VSV-ΔG pseudoviruses expressing the S proteins indicated in *A*. Points indicate individual NT_50_ values and bars the geometic means. Significance values compared with Wuhan are indicated by asterisks: *P < 0.03; **P < 0.002; ***P < 0.0002, ****P < 0.0001.

Several variant SARS-CoV-2 strains arose during the course of this study. As a prelude to more extensive experimentation, we evaluated the ability of serum from mice that had been vaccinated once or twice with the rMVA expressing the Wuhan S protein to neutralize pseudoviruses that express eight different variant or subvariant S proteins. The sequence changes relative to the Wuhan S, with those in the RBD indicated in red, are shown in Fig. 7*A*. The Alpha variant has only one amino acid substitution in the RBD relative to Wuhan. The E484K mutation is one of the three substitutions in the RBD of Beta and Gamma. The Delta Plus has one more mutation than Delta. Omicron has by far the most mutations within and outside of the RBD.

After a single vaccination, neutralization was significantly lower for all variants except Alpha and E484K, with the largest differences for Beta, Lambda, and Omicron (Fig. 7*B*). Following boosting, there were only small increases in the neutralization titers for Wuhan, Alpha, and E484K, but larger increases for the other variants, resulting in less but still significant differences from Wuhan (Fig. 7*B*).

## Discussion

In the first part of this report, we extended our previous investigations of the MVA-based SARS-CoV-2 vaccine rMVA-S_tri_ by determining the dose–response and the durability of binding and neutralizing antibodies. Protection against a respiratory infection with SARS-CoV-2 correlated with an NT_50_ of ∼100 or more, although titers of 100 times that were achieved with a single vaccination. Protection persisted for more than 6 mo despite a rate of decline of binding and neutralizing antibodies of ∼20% per month, similar to that reported following two doses of an mRNA vaccine in people (22). A concern regarding MVA, and other live vectors, is the potential of diminished boosting due to immunity to the vector. However, neutralizing antibody boosting occurred at both 3 wk and 6 mo after priming, with the highest response at the latter time. Unlike replicating virus vector vaccines that depend on spread to generate sufficient antigen, MVA is inoculated as a bolus of 2 × 10^7^ PFU that undergoes a single cycle of infection, which could diminish the negative impact of prior immunity.

In addition to neutralizing a pseudovirus expressing the homologous S, the serum from mice also neutralized pseudoviruses expressing a panel of variant and subvariant S proteins. Although each variant was neutralized, the efficacy was diminished for most with the lowest values for Beta and Omicron. Nevertheless, our cross-neutralization experiments in progress demonstrate protection upon challenge with variant viruses.

The focus of the present study was a comparison of IN and IM immunizations. Using a recombinant MVA encoding firefly Luc, luminescence was detected within 6 to 24 h in the head and chest following IN administration and at the site of inoculation following IM injection. As enzymatically active firefly Luc has a half-life of 3 to 4 h in mammalian cells (23), luminescence is a good indicator of active infection at the time of injecting luciferin. The decline in luminescence over 3 d and its localization reflects the inability of MVA to actively spread in mammalian cells, an important safety feature.

Having established the ability of MVA to infect the respiratory tract of mice, we compared S-binding IgG and IgA and T cells in lungs following priming and boosting by the IN and IM routes. IM vaccination elicited S-antigen–specific IgG and CD3^+^CD8^+^IFN-γ^+^ T cells in the lungs; however, only IN vaccination induced IgA and the T cell levels were significantly higher. The boosting of the IgA response by a second IN immunization is consistent with the establishment of tissue resident memory B and T cells (24, 25). IgA was also induced in the blood following IN inoculation. We found that even though one or two IM immunizations could not induce significant amounts of IgA in the lungs or blood, it could prime for a subsequent IN boost. Exactly how IM vaccination primes for an IgA response in the lungs and how IN immunization induces serum IgA are uncertain, but serum IgA has been found in individuals infected with SARS-CoV-2 (26, 27) and following mRNA vaccination (28–30).

To help distinguish between preventing and rapidly clearing infection, we determined the presence of sgRNAs as well as infectious SARS-CoV-2 in the nasal turbinates and lungs early after challenge. Following two IM vaccinations, SARS-CoV-2 in the lungs and nasal turbinates was undetectable by 4 d. However, reduced amounts of sgRNAs were detected in the nasal turbinates on day 2 but not day 5, indicating that virus replication was not entirely prevented by IM vaccination though subsequently cleared. In contrast, following two IN vaccinations, neither sgRNAs nor virus were detected in the turbinates or lungs on day 2, indicating that the infection was prevented or rapidly eliminated. Thus, the immune response following IM vaccination was sufficient to clear SARS-CoV-2 within several days despite the absence of IgA antibodies, whereas the response following IN vaccination either prevented infection or accelerated clearance, likely due to local IgA antibodies although IgG antibodies and CD8^+^ T cells may also contribute. Because of their polymeric structure, secretory IgA antibodies can potently neutralize SARS-CoV-2 (26).

Both the present report and one by Bošnjak et al. (31) demonstrate advantages of IN delivery of MVA-based SARS-CoV-2 vaccines, although there are some differences between the two studies. The MVA vectors from both laboratories expressed the Wuhan S, except our construct contained mutations that stabilize the prefusion form of S and prevent furin cleavage and endoplasmic reticulum retention. In both studies, IN delivery boosted IgA following IN and IM priming, but we detected IgA after a single IN vaccination, whereas the other study did not. We compared protection of the upper and lower respiratory pathways after IN, IM, IMxIN, and INxIN vaccinations of K18-hACE2 mice, whereas the other study examined protection of the lower respiratory pathway after IMxIN vaccination of Golden hamsters. In both studies, the lower respiratory tract of vaccinated animals was well protected. Our study further showed protection of the nasal turbinates with undetectable virus or sgRNAs after IN priming and boosting. A closer study to the present one is that of Hassan et al. (32), who reported that IN administration of a chimpanzee adenovirus vectored SARS-CoV-2 S vaccine also provided significant protection for the nasal turbinates and lungs in mice. Both the adenovirus and MVA vectors are replication-defective, providing an important safety feature.

MVA has an impressive safety profile as a smallpox vaccine. During the 1970s, 120,000 subjects, including children and high-risk subjects with allergy and skin diseases, were vaccinated in Germany with no reported adverse effects (33). More recently, MVA was shown to be safe and immunogenic when administered to hematopoietic stem cell transplant recipients (34–36) and HIV-infected subjects (37), and has been approved as a smallpox vaccine in the United States, Canada, and the European Union. While no rMVA-vectored vaccine has yet been approved, many are in clinical trials, including IM delivery of SARS-CoV-2 vaccines. The present report supports testing the delivery of MVA-vectored SARS-CoV-2 vaccines by the IN route with assessment of subsequent infection rates and transmission.

## Materials and Methods

A summary of materials and methods is outlined below. Detailed methods can be found in *SI Appendix*.

### Mice.

Five- to 6-wk-old female C57BL/6J and B6.Cg-Tg(K18-hACE2)2Prlmn/J mice were obtained from Taconic Biosciences and Jackson Laboratories, respectively.

### Vaccination.

IM vaccination was routinely performed as previously described (15), with 2 × 10^7^ PFU of rMVA-S_tri_ or with lower amounts indicated in the figure legends. For IN vaccination, mice were lightly sedated with isoflurane and 2 × 10^7^ PFU of rMVA-S_tri_ in 50 µL was administered.

### Challenge Virus.

SARS-CoV-2 USA-WA1/2019 from BEI resources (Ref# NR-52281) was propagated in Vero E6 cells and 10^5^ TCID_50_ was administered IN to mice that were lightly sedated with isoflurane.

### Analysis of S-Binding Antibodies.

Full-length S-binding antibodies were analyzed by ELISA using horseradish peroxidase-conjugated goat anti-mouse IgG or goat anti-mouse IgA. IgG and IgA endpoint titers were determined as fourfold above the average absorbance of those wells not containing primary antibody.

### Pseudovirus Neutralization Assays.

The CoV-2 S lentiviral pseudotype assay was carried out as described by Corbett et al. (38) and in our previous report (15). For the rVSVΔG pseudoviral neutralization assay, heat-inactivated serum, rVSVΔG pseudoviruses and anti–VSV-G hybridoma supernatant were incubated together and then added to Vero E6 cells expressing hTMPRSS2 and hACE2 and incubated for 20 h at 37 °C. GFP was measured by flow cytometry. NT_50_ values were calculated by plotting dose–response curves, normalized using the average of the no virus wells as 100% neutralization, and the average of the no serum wells as 0%. The limit of detection of 25 was determined by taking 1.96 SD of the mean titer of the control MVA samples.

### Isolation of Antibodies and Detection of T Cells from Lungs.

Procedures were similar to those described previously (21). Contaminating blood was removed by perfusion, lung tissue was digested with collagenase and DNase, and cells dissociated. Supernatants were clarified by high-speed centrifugation and used for antibody detection. Cells were pelleted and used for flow cytometric analysis of T cells after stimulation by peptide pools as described previously (15).

### Quantitation of Infectious SARS-CoV-2.

Dilutions of clarified homogenates of lungs and nasal turbinates were applied to Vero E6 cells. After 72 h, the plates were stained and the Reed–Muench method was used to determine the concentration at which 50% of the cells displayed a cytopathic effect (TCID_50_).

### Quantitation of SARS-CoV-2 sgRNAs.

RNA was extracted from cell homogenates of lungs and turbinates; contaminating DNA was removed and RNA was reverse-transcribed. SARS-CoV-2 sgS and sgN transcripts and 18S rRNA were quantified by ddPCR with specific primers using an automated droplet generator and droplet reader.

### Bioluminescence Imaging.

For IN vaccination of mice, 50 µL of 4 × 10^8^ PFU/mL was delivered into the right nostril. For IM vaccination, 50 µL of 2 × 10^8^ PFU/mL was injected into each hind leg of the mouse. To monitor vaccine virus infection in-vivo, an IVIS Lumina LT Series III System (Perkin-Elmer) was utilized. Mice were sedated and D-luciferin substrate was injected intraperitoneally. Luminescence was detected with an IVIS imager using the same exposure, binning and f-stop settings for each mouse.

### Safety and Ethics.

All experiments and procedures involving mice were approved under protocol LVD29E by the National Institute of Allergy and Infectious Diseases Animal Care and Use Committee according to standards set forth in the NIH guidelines, Animal Welfare Act, and US Federal Law. Euthanasia was carried out using carbon dioxide inhalation in accordance with the American Veterinary Medical Association Guidelines for Euthanasia of Animals (39). Experiments with SARS-CoV-2 were carried out under biosafety level-3 containment.

## Supplementary Material

Supplementary File

Supplementary File

## Data Availability

All data are included in the main text and supporting information. No data are deposited in an external source.
