# Supplementary material for: Intranasal inoculation of an MVA-based vaccine induces IgA and protects the respiratory tract of hACE2 mice from SARS-CoV-2 infection
Source: Proc Natl Acad Sci U S A. 2022 Jun 9;119(24):e2202069119. doi: 10.1073/pnas.2202069119 (PMC9214525; doi:10.1073/pnas.2202069119)
Supplement: Supplementary File [file pnas.2202069119.sapp.pdf]

## S1 APPENDIX. MATERIALS AND METHODS

**Mice.** Five- to six-week-old female C57BL/6ANTac and B6.Cg-Tg(K18-hACE2)2Prlmn/J mice were obtained from Taconic Biosciences and Jackson Laboratories, respectively. Typically, 3-5 mice were housed per sterile, ventilated microisolator cage in an ABSL-2 or ABSL-3 facility.

**MVA viruses and cells.** Sucrose gradient purified rMVA-$S_{tri}$ (1), MVA/Luc (2), and MVA clone 1 of Wyatt et al. (3) were prepared as described previously. Vero E6 cells (ATCC CRL-1586) were maintained in Dulbecco's Modified Eagle Medium (DMEM) supplemented with 8% heat-inactivated fetal bovine serum (FBS), 2 mM L-glutamine, 10 U/ml penicillin, and 10 µg/ml streptomycin.

**Vaccination.** Viruses used for vaccination were thawed, dispersed by sonication, and 10-fold serial dilutions were made in PBS containing 0.05% BSA, resulting in concentrations ranging from $2x10^8$ to $2x10^4$ PFU/ml. The rMVAs in 50 µl were injected IM into each hind leg of the mouse. For IN vaccination, mice were lightly sedated with isoflurane and 50 µl of rMVAs administered.

**Infection with SARS-CoV-2**. SARS-CoV-2 USA-WA1/2019 from BEI resources (Ref# NR-52281) was propagated in Vero E6 cells in a BSL-3 laboratory by Bernard Lafont of the NIAID SARS-CoV-2 Virology Core laboratory. The clarified culture medium was titrated on Vero E6 cells and the $TCID_{50}$ was determined by the Reed-Muench method. An aliquot consisting of $10^5$ $TCID_{50}$ of SARS-CoV-2 in 50 µl was administered IN to mice that were lightly sedated with

isoflurane. After infection, the weights and morbidity/mortality status were assessed and recorded daily for up to 14 days.

**Detection of S-binding IgG and IgA antibodies by ELISA.** SARS-CoV-2 (2019-nCoV) spike (S1+S2 ECD protein, Sino Biologicals) was diluted in phosphate buffered saline (PBS) to a concentration of 1 µg/ml. MaxiSorp 96-well flat-bottom plates (Thermo Fisher) were filled with 100 µl of diluted S protein (0.1 µg/well) and incubated overnight at 4$^{o}$C. After adsorption, wells were washed three times with 250 µl PBS + 0.05% Tween-20 (PBS-T, Accurate Chemical). Plates were blocked for 2 h at room temperature with 200 µl PBS-T + 5% nonfat milk and subsequently washed three times with PBS-T prior to incubation with a series of eight 4-fold dilutions of mouse sera or lung homogenate supernatants for 1 h at room temperature. To detect S-specific IgG or IgA antibodies, plates were washed three times with PBS-T and incubated with horseradish peroxidase (HRP)-conjugated goat anti-mouse IgG (H+L) (Thermo Fisher) or HRP-conjugated goat anti-mouse IgA (Southern Biotech) for 1 h at room temperature. After incubation with either detection antibody, plates were washed three times with PBS-T and 100 µl of pre-warmed SureBlue TMB substrate (SeraCare) was added to the plate for 10 min at room temperature. To stop the colorimetric reaction, 100 µl of 1N sulfuric acid was added to each well and absorbance was measured at $A_{450}$ and $A_{650}$ using a Synergy H1 plate reader with Gen5 analysis software (Agilent Technologies). IgG and IgA endpoint titers were determined as 4-fold above the average absorbance of those wells not containing primary antibody.

**Preparation of lung tissues**. Mice were euthanized via cervical dislocation and 12 ml of PBS were injected into the right ventricle to remove contaminating blood. Lungs were excised and

placed in 2 ml of cold Tissue Storage Solution (Miltenyi Biotec). Individual lobes were placed in gentleMACS C-tubes (Miltenyi Biotec) containing collagenase and DNase provided in the Mouse Lung Dissociation Kit (Miltenyi Biotec). Tissues were homogenized using a gentleMACS Octo Dissociator with Heaters (Miltenyi Biotec) and clarified by centrifugation at 300xg for 10 min at 4$^{o}$C. Supernatants were further clarified by high-speed centrifugation and used for detection of antibodies. Cell pellets were suspended in autoMACS Rinsing Solution containing 0.5% bovine serum albumin (BSA) and filtered through a 70 µm cell strainer. Cells were pelleted by centrifugation at 300xg for 10 min at 4$^{o}$C and suspended in 1X Red Blood Cell Lysis Buffer (Miltenyi Biotec). After removal of red blood cells, the remaining cells were washed three times in complete RPMI medium (cRPMI, supplemented with 10% heat-inactivated FBS, 2 mM L-glutamine, 10 U/ml penicillin, 10 µg/ml streptomycin, and 2 mM HEPES). After the last wash, cells were counted using a T4 Automated Cellometer (Nexcelom) and suspended in cRPMI at $5x10^{6}$ cells/ml.

**Stimulation and flow cytometric analysis of lung T cells.** Approximately $10^{6}$ lung cells in 0.2 ml of cRPMI from individual mice were stimulated with 1 µM of two different peptide pools, derived from a complete set of SARS-CoV-2 Spike peptides obtained from BEI Resources (NR-52402), and previously shown to induce IFNγ production in splenocytes isolated from rMVA-$S_{tri}$ vaccinated mice (1). The peptides used to make the pools contained SARS-CoV-2 Spike peptides 32-41 (Pool 4) and peptides 61, 64, 67 (Pool 7). After stimulation for 1.5 h at 37$^{o}$C, 2 µg of brefeldin A was added to each sample for an additional 4-5 h. To block Fc receptors, anti-CD16/32 (Clone 2.4G2, a gift from Jack Bennink, NIAID) was added prior to performing surface staining with anti-mouse CD3-FITC, anti-mouse CD4-PE and anti-CD8-PerCP-Cy5.5 for

1 h. Cells were then fixed and permeabilized with Cytofix/Cytoperm solution (BD Biosciences) and stained with IFNγ-APC and fixed with 2% paraformaldehyde. From 10,000 to 70,000 events were acquired on a FACSCelesta cytometer running FACSDiva software (BD Biosciences) and analyzed with FlowJo software.

**Pseudovirus neutralization assays.** Lentivirus- and VSV-based neutralization assays were used as indicated in Fig. Legends. The CoV-2 lentiviral pseudotype assay was carried out as described by Corbett et al. (4) and in our previous report (1). Luciferase units (RLU) were read on a luminometer and the NT50 values were calculated using Prism to plot dose-response curves, normalized using the average of the no virus wells as 100% neutralization, and the average of the no serum wells as 0%. The limit of detection (LOD) of 50 NT50 was determined by taking 1.96 standard deviation of the mean titer of the control MVA samples.

For construction of VSV-based pseudoviruses, BHK-21 cell lines expressing SARS-CoV-2 codon optimized spike with a truncation of the 19 C-terminal amino acids were prepared using the Sleeping Beauty transposon system (5). Full length codon-optimized SARS-CoV-2 spike protein gene (Wuhan-1, GenBank MN908947.3) in pVRC8400 was received from the NIAID Vaccine Research Center. Primers were designed to insert the spike gene with truncation of the DNA encoding the 19 C-terminal amino acids into pSBBi-Pur from Addgene #60523 (5). BHK-21 cells (ATCC#CCL-10) were transfected with pSBBi-Pur spike plasmid and pCMV (CAT) T7-SB100 expressing hyperactive transposase (Addgene #34879) (6). Cells were selected with puromycin, and expression of spike verified by Western blotting and flow cytometry with spike-specific antibodies.

To produce the pseudovirus, BHK-21 cells expressing SARS-CoV-2 spike were infected at a multiplicity of 3 PFU with rVSVΔG expressing green fluorescent protein (GFP) provided by Ivan Kosik, NIAID (7). At 24 h post infection the medium was collected, and cell debris pelleted by low-speed centrifugation (1200 rpm, 10 min). The supernatant was filtered (0.45 µm) and single use aliquots stored at -80ºC. Pseudoviruses were titrated on VeroE6 cells expressing human TMPRSS2 and hACE2 obtained from Jaroslav Holly, NIAID.

For the rVSVΔG pseudoviral neutralization assay, serial dilutions of heat-inactivated sera were incubated with rVSVΔG pseudoviruses and anti-VSV-G I1 hybridoma supernatant (ATCC# CRL-2700) for 45 min at 37ºC. The mixture was then added to VeroE6 cells expressing hTMPRSS2 and hACE2 and incubated for 20 h at 37ºC. The cells were fixed in 2% paraformaldehyde and GFP measured by flow cytometry. NT50 values were calculated using Prism (Graphpad) to plot dose-response curves, normalized using the average of the no virus wells as 100% neutralization, and the average of the no serum wells as 0%. The limit of detection (LOD) of 25 was determined by taking 1.96 standard deviation of the mean titer of the control MVA samples.

**Quantitation of infectious SARS-CoV-2.** Lungs and nasal turbinates were homogenized, cleared of debris by centrifugation at 3800xg for 10 min and serial 10-fold dilutions were applied in quadruplicate to Vero E6 cells in DMEM+Glutamax (ThermoFisher) supplemented with 2% heat-inactivated FBS and 1% Antibiotic-Antimycotic in 96-well microtiter plates. After 72 h, the plates were stained with crystal violet and the Reed-Muench method was used to determine the concentration at which 50% of the cells displayed a cytopathic effect ($TCID_{50}$).

**Quantitation of SARS-CoV-2 sgRNAs.** RNA was extracted from homogenates of lungs and turbinates using Trizol; contaminating DNA was removed and RNA was reverse-transcribed. SARS-CoV-2 sgS and sgN transcripts and 18S rRNA were quantified by ddPCR with specific primers using an automated droplet generator and droplet reader (BioRad).

**Bioluminescence imaging.** Purified MVA/Luc was diluted in PBS containing 0.05% BSA. Prior to inoculation, C57BL/6 mice were lighted sedated by inhalation of isoflurane gas. For IN vaccination of mice, 50 µl of $4x10^8$ PFU/ml was delivered into the right nostril. For IM vaccination, 50 µl of $2x10^8$ PFU/ml was injected into each hind leg of the mouse. To monitor vaccine virus infection in-vivo, an IVIS Lumina LT Series III System (Perkin Elmer) was utilized. Mice were sedated and Xenolight D-luciferin substrate (Perkin Elmer) was injected IP at 150 µg/g body weight. After 10 min, luminescent images were collected using the same exposure, binning and f-stop settings for each mouse. Images were acquired and analyzed using Living Image Software (Perkin Elmer). To determine photon flux, regions of interest were drawn around different areas of the body and light emission was measured in photons/sec/$cd^2$/sr.

## REFERENCES

1. R. K. Liu *et al.*, One or two injections of MVA-vectored vaccine shields hACE2 transgenic mice from SARS-CoV-2 upper and lower respiratory tract infection. *Proceedings of the National Academy of Sciences of the United States of America* **118** (2021).
2. L. S. Wyatt, W. Xiao, J. L. Americo, P. L. Earl, B. Moss, Novel Nonreplicating Vaccinia Virus Vector Enhances Expression of Heterologous Genes and Suppresses Synthesis of Endogenous Viral Proteins. *Mbio* **8** (2017).
3. L. S. Wyatt, P. L. Earl, L. A. Eller, B. Moss, Highly attenuated smallpox vaccine protects mice with and without immune deficiencies against pathogenic vaccinia virus challenge. *Proc. Nat. Acad. Sci. USA* **101**, 4590-4595 (2004).
4. K. S. Corbett *et al.*, SARS-CoV-2 mRNA vaccine design enabled by prototype pathogen preparedness. *Nature* 10.1038/s41586-020-2622-0 (2020).
5. E. Kowarz, D. Loscher, R. Marschalek, Optimized Sleeping Beauty transposons rapidly generate stable transgenic cell lines. *Biotechnol J* **10**, 647-653 (2015).

6. L. Mates *et al.*, Molecular evolution of a novel hyperactive Sleeping Beauty transposase enables robust stable gene transfer in vertebrates. *Nat Genet* **41**, 753-761 (2009).
7. J. Woodford *et al.*, Severe Acute Respiratory Syndrome Coronavirus 2 Seroassay Performance and Optimization in a Population With High Background Reactivity in Mali. *J Infect Dis* **224**, 2001-2009 (2021).
